# Re-evaluating the role of adjuvant chemotherapy after neoadjuvant chemotherapy in resected gastric cancer

**DOI:** 10.1093/oncolo/oyag384

**Published:** 2026-10-03

**Authors:** Aravind Sreeram, Celine Yeh, Nicolas Toumbacaris, Marinela Capanu, Shoji Shimada, Ping Gu, Yelena Y Janjigian, David H Ilson, Vivian E Strong, Geoffrey Y Ku

**Affiliations:** Department of Surgery, Memorial Sloan Kettering Cancer Center, New York, NY 10065, United States; Department of Medicine, Memorial Sloan Kettering Cancer Center, New York, NY 10065, United States; Department of Epidemiology and Biostatistics, Memorial Sloan Kettering Cancer Center, New York, NY 10065, United States; Department of Epidemiology and Biostatistics, Memorial Sloan Kettering Cancer Center, New York, NY 10065, United States; Department of Surgery, Memorial Sloan Kettering Cancer Center, New York, NY 10065, United States; Department of Medicine, Memorial Sloan Kettering Cancer Center, New York, NY 10065, United States; Department of Medicine, Memorial Sloan Kettering Cancer Center, New York, NY 10065, United States; Department of Medicine, Memorial Sloan Kettering Cancer Center, New York, NY 10065, United States; Department of Surgery, Memorial Sloan Kettering Cancer Center, New York, NY 10065, United States; Department of Medicine, Memorial Sloan Kettering Cancer Center, New York, NY 10065, United States

**Keywords:** stomach neoplasms, neoadjuvant therapy, event-free survival, retrospective studies, lymph node ratio

## Abstract

**Background:**

Perioperative chemotherapy is the standard of care for resectable gastric cancer (GC), but many patients do not complete adjuvant chemotherapy (AC). It remains unclear whether AC following neoadjuvant chemotherapy (NAC) improves survival. We sought to assess differences in survival between patients with resectable GC who did vs did not receive AC after NAC and surgery.

**Methods:**

A retrospective study was conducted at a tertiary cancer center to evaluate survival differences in patients who did vs did not receive AC. Patients with resected GC who received NAC were included.

**Results:**

Of 339 patients, 264 (78%) received AC. There were no significant differences in median event-free survival (EFS) (7.0 vs. 7.5 years, *P* = .089) or overall survival (OS) (10 vs. 7.8 years, *P* = .11) between AC and non-AC groups. In the node-negative (ypN−) cohort (*n* = 169), there was no association between AC receipt and EFS (12 vs. 10 years, *P* = .7) or OS (12 vs. 10 years, *P* = .5). In the node-positive (ypN+) cohort (*n* = 170), AC receipt was associated with longer EFS (4.8 vs. 2.7 years, *P* = .031) but not OS (5.5 vs. 2.8 years, *P* = .071). High-risk patients based on lymph node ratio (LNR > 0.0685; *n* = 112) who received AC (*n* = 87; 78%) had longer EFS (*P* = .014) and OS (*P* = .033). Finally, AC receipt was associated with longer EFS (*P* = .014) and OS (*P* = .024) in those with incomplete/poor response to NAC (Becker tumor regression grade [TRG] 2-3).

**Conclusions:**

AC following NAC was not associated with improved survival in resected ypN- GC. High LNR identified a ypN + subgroup in which AC receipt was associated with longer EFS and OS. In patients with incomplete/poor response to NAC (TRG 2-3), AC receipt was associated with longer EFS and OS.

Implications for practice Less than half of patients who receive neoadjuvant chemotherapy and undergo gastrectomy for locally advanced gastric cancer are ultimately able to complete planned adjuvant chemotherapy. We investigated whether the receipt of adjuvant chemotherapy in patients who have completed neoadjuvant chemotherapy is associated with clinical benefit. We further identified clinical and pathologic characteristics that may confer benefit from adjuvant chemotherapy pending prospective validation. In this retrospective, single-center analysis, adjuvant chemotherapy was associated with a significant event-free survival benefit in patients with ypN + disease, but not in the overall or ypN − cohorts. Within the ypN + cohort, high lymph node ratio at time of surgery represents a high-risk group that may derive benefit from adjuvant chemotherapy. Finally, incomplete/poor pathologic response to neoadjuvant chemotherapy is associated with benefit from adjuvant chemotherapy.

## Introduction

Gastric cancer (GC) ranks as the fifth most common cancer globally and the third leading cause of cancer-related death.^1^^,^^2^ In Western countries, perioperative chemotherapy and surgery for locally advanced/resectable disease are recommended based on an overall survival (OS) benefit initially established by the MAGIC study over surgery alone.^3^ Although biomarkers such as pathologic complete response^4^ and lymph node negativity (ypN−)^5^ have been associated with improved outcomes, both ESMO^6^ and NCCN guidelines^7^ recommend completion of adjuvant chemotherapy (AC) for all patients who initiate preoperative chemotherapy regardless of pathologic findings at time of surgery.

Despite this recommendation, many patients are unable to initiate or complete AC for many reasons, including disease progression, postoperative complications and previous chemotherapy toxicity.^3^^,^^8^ In the MAGIC study, only 66% of patients initiated and 42% completed all planned AC.^3^ The FLOT4 study, which has since established perioperative 5-fluorouracil, leucovorin, oxaliplatin, and docetaxel (FLOT) as the current standard of care, reported that only 60% and 46%, respectively, initiated and completed AC.^8^

Numerous retrospective studies have explored whether the receipt of AC is associated with improved survival but the conclusions have been inconsistent.^9–12^ We present a single-center retrospective analysis of patients with resectable GC treated at Memorial Sloan Kettering Cancer Center (MSKCC) with NAC followed by curative-intent gastrectomy. Our objectives were to: (1) compare OS and event-free survival (EFS) between patients who received perioperative chemotherapy versus NAC only, (2) identify reasons underlying noninitiation/completion of AC, (3) identify patient subgroups for whom efforts should be made to administer AC, or conversely, (4) identify patient subgroups for whom AC may be omitted due to clear lack of survival benefit. To maintain a more homogeneous cohort of gastric primary tumors, we excluded tumors with predominant gastroesophageal junction involvement, defined as >50% of the tumor localized above the gastric cardia. We comprehensively report details of NAC and AC, including regimen, number of cycles planned and completed, intention to administer AC, and reasons for non-initiation/completion of AC. To our knowledge, this is the first study to assess the impact of AC receipt on survival in a North American population while integrating detailed information surrounding perioperative chemotherapy and applying rigorous exclusion criteria to enhance homogeneity of the study cohort.

## Methods

A retrospective cohort study was performed after MSKCC Institutional Review Board (IRB) approval (Protocol #19-111). Given the retrospective and de-identified nature of the study, the requirement for informed consent was waived by the IRB. Six hundred and ninety patients treated at MSKCC for resectable GC between January 1, 2006, and December 31, 2022 were identified from an internal database, followed by manual review of medical records. Patients were excluded from the analysis if they: (1) had known mismatch repair-deficient/microsatellite instability-high (dMMR/MSI-H) disease (*n* = 66); (2) had involvement of the gastroesophageal junction (GEJ) where the bulk of the tumor (>50%) localized to above the gastric cardia (*n* = 100); (3) had M1 disease (*n* = 96); (4) received neoadjuvant radiotherapy (*n* = 7); (5) received NAC with total neoadjuvant intent, defined as >50% of total planned cycles of chemotherapy given prior to surgery (eg, >4 cycles of FLOT, >3 cycles of ECF-like regimens) (*n* = 49); (6) received <66% of planned cycles of NAC (*n* = 4); (7) did not have complete chemotherapy data (*n* = 26); (8) died within 30 days of surgery (*n* = 2); or (9) received AC >4 months after surgery (*n* = 1) (Figure S1, see online supplementary material for a color version of this figure). Data collection occurred between December 1, 2023, and March 18, 2024. This study was conducted and reported in accordance with the Strengthening the Reporting of Observational Studies in Epidemiology (STROBE) guidelines.

Baseline staging included PET/CT scans and diagnostic laparoscopy and, when appropriate, endoscopic ultrasound. Institutional practice regarding perioperative chemotherapy followed NCCN guidelines.^7^ All patients had resectable GC, received NAC, and were recommended to receive AC either as standard care (*n* = 325) or on a clinical trial (*n* = 14). Surgery was performed between 2 and 8 weeks following the last administration of NAC and included D2 lymphadenectomy. Pathologic staging was performed according to the AJCC ypTNM classification and by a dedicated subspecialty pathologist.^13^ Patients who received ≥1 cycle of AC within 4 months after surgery constituted the AC group. Patients who did not receive any AC constituted the non-AC group. Pathologic response to NAC was quantified using the Becker tumor regression grade (TRG).^14^

Patient clinicopathologic characteristics were summarized using medians and interquartile ranges (IQR) for continuous variables, and counts and percentages for categorical variables. Differences in clinicopathologic characteristics were compared with the Wilcoxon rank-sum test for continuous variables and Pearson’s chi-squared test or Fisher’s exact test for categorical variables. The primary endpoints were EFS and OS. EFS was calculated from date of surgery to date of progression, recurrence, or death, whichever occurred first. OS was calculated from date of surgery to date of death. Patients were censored at the date of last available follow-up. OS and EFS were estimated using the Kaplan-Meier approach and log-rank test *P* values were used to quantify differences in survival curves. Univariable and multivariable Cox models were built to analyze the association between clinicopathologic factors and OS and EFS. A landmark analysis at 60 days post-surgery (selected to exceed the maximum observed length of stay [LOS]) was performed to evaluate the associations between postoperative LOS and survival outcomes and to address potential immortal-time bias. A lymph node ratio (LNR) cutoff was calculated using maximally selected log-rank statistics for the outcome of EFS.^15^ Variables with global or individual *P*-values < .05 in univariable analysis were included in multivariable models. AC receipt was retained in all multivariable models a priori, irrespective of its statistical significance in the univariable analysis, because it represented the primary exposure of interest. All statistical tests were two-tailed with a type I error rate (*α*) of 0.05. Statistical analyses were conducted using R (version 4.3.3, R Core Development Team, Vienna, Austria).

## Results

### Patient and disease characteristics

Three hundred thirty-nine patients were included. Median (IQR) age at surgery was 64 years (54-72). Sixty percent were male, and 66% were White. One hundred forty-one patients (42%) underwent total/proximal gastrectomy; 336 patients (99%) underwent R0 resection. Among resected tumors, 64% were ypT3-4, 50% were ypN+, and 52% exhibited >50% viable tumor following NAC (Becker TRG 3). The median (IQR) number of lymph nodes examined was 27 (20-37). Two hundred sixty-four patients (78%) received ≥1 cycle of AC. Compared to those who did not receive AC, patients who received AC were younger (62 vs. 68 years, *P* < .001), had fewer dissected lymph nodes (26 vs. 31 nodes, *P* = .003), and had shorter postoperative LOS (6 vs. 7 days, *P* < .001; Table 1). Patients who received AC also exhibited different trends in Becker TRG and ypT distributions, though these were not statistically significant.

**Table 1.** Patient and tumor characteristics stratified by AC receipt.

Characteristic	Overall (*N* = 339)^a^	Non-AC (*N* = 75)^a^	AC (*N* = 264)^a^	*P*-value^b^
**Age (years)**	64 (54, 72)	68 (61, 75)	62 (53, 71)	**<.001**
**Male sex**	205 (60%)	51 (68%)	154 (58%)	.13
**Race**				>.9
** White**	225 (66%)	51 (68%)	174 (66%)	
** Asian**	55 (16%)	12 (16%)	43 (16%)	
** Black**	34 (10%)	7 (9.3%)	27 (10%)	
** Other/unknown**	25 (7.4%)	5 (6.7%)	20 (7.6%)	
**Location**				.085
** Lower**	127 (37%)	20 (27%)	107 (41%)	
** Middle**	119 (35%)	28 (37%)	91 (34%)	
** Upper**	90 (27%)	26 (35%)	64 (24%)	
** Whole/diffuse**	3 (0.9%)	1 (1.3%)	2 (0.8%)	
**Lauren type**				.3
** Diffuse**	100 (30%)	18 (25%)	82 (32%)	
** Intestinal**	156 (47%)	34 (47%)	122 (47%)	
** Mixed**	75 (23%)	21 (29%)	54 (21%)	
** Unknown**	8	2	6	
**Surgery type**				.071
** Total/proximal gastrectomy**	141 (42%)	38 (51%)	103 (39%)	
** Other gastrectomy**	198 (58%)	37 (49%)	161 (61%)	
**Becker TRG**				.061
** 1a**	27 (8.0%)	10 (13%)	17 (6.4%)	
** 1b**	18 (5.3%)	4 (5.3%)	14 (5.3%)	
** 2**	118 (35%)	18 (24%)	100 (38%)	
** 3**	176 (52%)	43 (57%)	133 (50%)	
**ypT**				.057
** 0**	28 (8.3%)	10 (13%)	18 (6.8%)	
** 1**	49 (14%)	14 (19%)	35 (13%)	
** 2**	48 (14%)	9 (12%)	39 (15%)	
** 3**	141 (42%)	22 (29%)	119 (45%)	
** 4**	73 (22%)	20 (27%)	53 (20%)	
**ypN**				.8
** N0**	169 (50%)	37 (49%)	132 (50%)	
** N1**	66 (19%)	12 (16%)	54 (20%)	
** N2**	58 (17%)	14 (19%)	44 (17%)	
** N3**	46 (14%)	12 (16%)	34 (13%)	
**Dissected LNs**	27 (20, 37)	31 (23, 45)	26 (20, 35)	**.003**
**Positive LNs**	1.0 (0.0, 4.0)	1.0 (0.0, 5.0)	0.5 (0.0, 3.0)	.7
**PNI**	173 (51%)	36 (48%)	137 (52%)	.6
**LVI**	159 (47%)	41 (55%)	118 (45%)	.13
**Negative margins**	336 (99%)	74 (99%)	262 (99%)	.5
**Post-Op LOS (days)**	6.0 (5.0, 8.0)	7.0 (5.0, 11.0)	6.0 (5.0, 7.0)	**<.001**

Abbreviations: LN, lymph node; LOS, length of stay; LVI, lymphovascular invasion; PNI, perineural invasion.

a *n* (%); median (Q1, Q3).

b Fisher’s exact test; Pearson’s chi-squared test; Wilcoxon rank sum test. Significant *P*-values are bolded.

### Chemotherapy regimens

All patients completed at least 66% of planned cycles of NAC. NAC regimens were categorized as anthracycline + platinum + fluoropyrimidine (56%), platinum + fluoropyrimidine (25%), or taxane + platinum + fluoropyrimidine (19%). AC regimens were categorized as anthracycline + platinum + fluoropyrimidine (44%), fluoropyrimidine + platinum (26%), fluoropyrimidine + platinum + taxane (14%), fluoropyrimidine alone (11%), fluoropyrimidine + taxane (0.4%), or other (4.5%). Detailed information on chemotherapy regimens can be found in Table S1. Two hundred sixty-four patients (78%) received ≥1 cycle of AC, of whom 208 (78.8%) completed all planned cycles. Eleven patients (3.2%) underwent a change in chemotherapy regimen from the neoadjuvant to adjuvant setting due to significant toxicity with NAC (*n* = 2), poor PET and/or pathologic response to NAC (*n* = 6), change in standard-of-care since NAC (*n* = 1), neuroendocrine component detected on surgical pathology (*n* = 1), and for unclear reasons (*n* = 1).

### Reasons for not initiating/completing AC

Reasons for not initiating AC (*n* = 75) included postoperative complications (20%), poor performance status (16%), minimal pathologic response to NAC (14.7%), patient choice (14.7%), significant toxicity in the neoadjuvant setting (9.3%), no anticipated additional benefit per the treating physician (8%), and pathologic complete or near-complete response (6.7%); the reason was unknown in 10.7% of patients. Among patients who initiated AC (*n* = 264), 208 (78.8%) completed all planned therapy. Reasons for AC cessation or non-completion included toxicity (13.6%), postoperative complications (1.9%), disease recurrence (0.8%), and patient choice (0.8%); the reason was unknown in 4.2% of cases (Table S1).

### Survival analyses by receipt of AC in the overall cohort

The median follow-up time was 6.36 years (95% CI 5.87-7.05). The overall cohort had a median EFS and OS of 7.0 years (95% CI 6.4-9.7) and 9.5 years (95% CI 6.8-12), respectively, and estimated 5-year EFS and OS rates of 60% (95% CI 55%-66%) and 63% (95% CI 58%-69%), respectively. There were no significant differences in EFS (7.0 vs. 7.5 years, *P* = .089) or OS (10 vs. 7.8 years, *P* = .11) between patients who received AC and those who did not. To mitigate the prospect of minimal AC receipt skewing our results, as an exploratory analysis, we compared EFS and OS among patients receiving less than 50% of their AC cycles or no AC against patients who received at least 50% of planned AC cycles, and results were unchanged (Figure S2, see online supplementary material for a color version of this figure).

On univariable analysis of OS, higher ypT stage (*P* < .001), higher ypN stage (*P* < .001), and higher Becker TRG (*P* = .017) were associated with shorter OS, while Asian race was associated with longer OS (*P* = .014). Receipt of AC was not associated with longer OS (*P* = .11). On multivariable analysis, Asian race remained associated with longer OS (*P* = .005), while higher ypN stage remained associated with shorter OS (*P* < .001). Receipt of AC remained non-significant (*P* = .083). Cox regression analyses of EFS and OS are summarized in Tables S2 and S3.

To minimize potential bias caused by confounding variables, we performed 1:1 propensity score matching (PSM) based on age, AJCC stage, tumor location, and Clavien-Dindo score (Table S4 and Figure S3, see online supplementary material for a color version of this figure). Seventy patients were assigned to each cohort (AC and non-AC). After PSM, there was no significant difference in EFS (HR 1.35 [0.84-2.15]; *P* = .2) or OS (HR 1.36 [0.82-2.24]; *P* = .2) between patients who received AC and those who did not.

### Subgroup analysis by ypN status

To identify patients who would most likely benefit from AC, we performed subgroup analyses on the ypN+ (*n* = 170) and ypN- (*n* = 169) cohorts. Patients in the ypN + group were more likely to have higher TRG, ypT, ypN, perineural invasion, and lymphovascular invasion (all *P* < .001; Table 2). In each of the ypN + and ypN- cohorts, 132 patients received AC.

**Table 2.** Patient and tumor characteristics stratified by ypN status.

Characteristic	Overall (*N* = 339)^a^	ypN− (*N* = 169)^a^	ypN+ (*N* = 170)^a^	*P*-value^b^
**Age (years)**	64 (54, 72)	64 (55, 71)	64 (53, 73)	.8
**Male sex**	205 (60%)	111 (66%)	94 (55%)	.051
**Race**				.2
** White**	225 (66%)	112 (66%)	113 (66%)	
** Asian**	55 (16%)	29 (17%)	26 (15%)	
** Black**	34 (10%)	20 (12%)	14 (8.2%)	
** Other/unknown**	25 (7.4%)	8 (4.7%)	17 (10%)	
**Location**				.5
** Lower**	127 (37%)	70 (41%)	57 (34%)	
** Middle**	119 (35%)	54 (32%)	65 (38%)	
** Upper**	90 (27%)	44 (26%)	46 (27%)	
** Whole/diffuse**	3 (0.9%)	1 (0.6%)	2 (1.2%)	
**Lauren type**				.3
** Diffuse**	100 (30%)	45 (28%)	55 (33%)	
** Intestinal**	156 (47%)	83 (51%)	73 (43%)	
** Mixed**	75 (23%)	34 (21%)	41 (24%)	
** Unknown**	8	7	1	
**Surgery type**				.3
** Total/proximal gastrectomy**	141 (42%)	66 (39%)	75 (44%)	
** Other gastrectomy**	198 (58%)	103 (61%)	95 (56%)	
**Received AC**	264 (78%)	132 (78%)	132 (78%)	>.9
**Becker TRG**				**<.001**
** 1a**	27 (8.0%)	27 (16%)	0 (0%)	
** 1b**	18 (5.3%)	11 (6.5%)	7 (4.1%)	
** 2**	118 (35%)	65 (38%)	53 (31%)	
** 3**	176 (52%)	66 (39%)	110 (65%)	
**ypT**				**<.001**
** 0**	28 (8.3%)	27 (16%)	1 (0.6%)	
** 1**	49 (14%)	37 (22%)	12 (7.1%)	
** 2**	48 (14%)	24 (14%)	24 (14%)	
** 3**	141 (42%)	59 (35%)	82 (48%)	
** 4**	73 (22%)	22 (13%)	51 (30%)	
**ypN**				**<.001**
** 0**	169 (50%)	169 (100%)	0 (0%)	
** 1**	66 (19%)	0 (0%)	66 (39%)	
** 2**	58 (17%)	0 (0%)	58 (34%)	
** 3**	46 (14%)	0 (0%)	46 (27%)	
**Dissected LNs**	27 (20, 37)	26 (20, 36)	28 (21, 41)	.2
**Positive LNs**	1.0 (0.0, 4.0)	0.0 (0.0, 0.0)	4.0 (1.0, 7.0)	**<.001**
**PNI**	173 (51%)	63 (37%)	110 (65%)	**<.001**
**LVI**	159 (47%)	39 (23%)	120 (71%)	**<.001**
**Negative margins**	336 (99%)	169 (100%)	167 (98%)	.4
**Post-Op LOS (days)**	6.0 (5.0, 8.0)	6.0 (5.0, 7.0)	6.0 (5.0, 8.0)	.069

Abbreviations: LN, lymph node; LOS, length of stay; LVI, lymphovascular invasion; PNI, perineural invasion.

a *n* (%); median (Q1, Q3).

b Pearson’s chi-squared test; Wilcoxon rank sum test; Fisher’s exact test. Significant *P*- values are bolded.

In the ypN + cohort, patients who received AC had significantly longer EFS than those who did not receive AC (4.8 vs. 2.7 years, *P* = .031) (Figure 1A). Estimated 5-year EFS was 48% (95% CI 40%-58%) and 29% (17%-49%) in the AC and non-AC groups, respectively.

**Figure 1.**
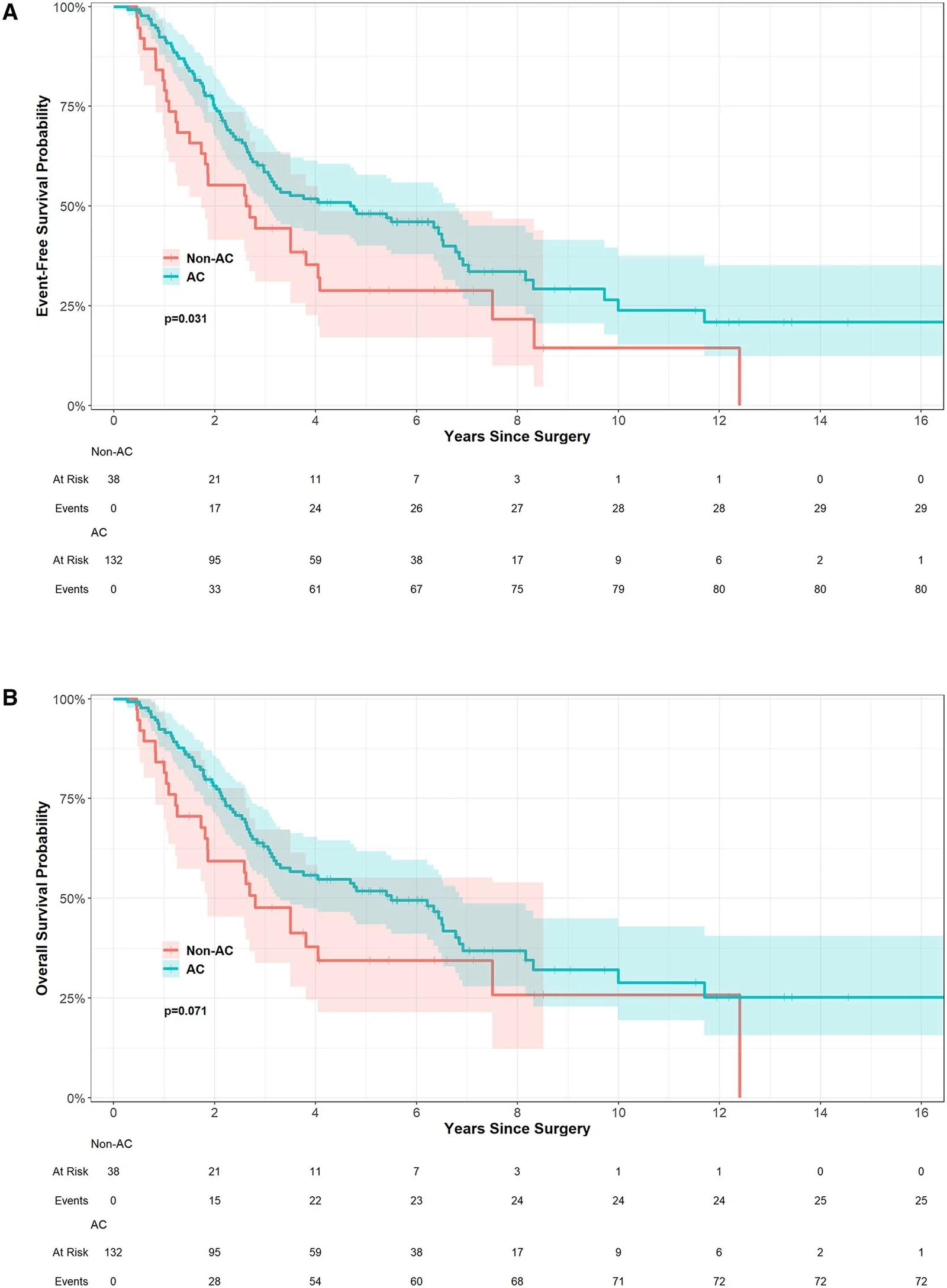
Kaplan-Meier analyses of (A) event-free and (B) overall survival among ypN+ patients.

However, there was only a non-statistically significant trend toward improved OS for patients who received AC versus those who did not (5.5 vs. 2.8 years, *P* = .071) (Figure 1B). Estimated 5-year OS was 52% (95% CI 43%-62%) and 34% (22%-55%) in the AC and non-AC groups, respectively.

On univariable analysis of EFS in the ypN + cohort, diffuse histologic type compared to intestinal (*P* = .006) and ypN3 stage compared to ypN1 (*P* < .001) were associated with shorter EFS, while lower ypT stage compared to ypT4 (*P* < .01) and receipt of AC was associated with longer EFS (*P* = .032). On multivariable analysis of the ypN + cohort, while higher ypT (*P* = .045) and ypN (*P* = .002) stages remained associated with shorter EFS, the association between receipt of AC and longer EFS was no longer significant (*P* = .2) (Table 3). On univariable analysis of OS in the ypN + cohort, diffuse histologic type compared to intestinal type (*P* = .037), ypT4 compared to ypT1-3 stage (*P* < .05), and ypN3 compared to ypN1-2 stage (*P* < .001) were associated with shorter OS, while receipt of AC was not associated with a significant difference in OS (*P* = .073). On multivariable analysis, only ypN3 stage remained associated with shorter OS (*P* = .002), and the association between receipt of AC and OS remained non-significant (*P* = .3) (Table S5). Of note, there was only 1 patient in the ypN + cohort with ypT0 disease and 12 patients with ypT1 disease, limiting analysis of these subgroups in these models.

**Table 3.** Univariable and multivariable analyses for EFS in the ypN + cohort.

Characteristic	Univariable analysis					Multivariable analysis				
	*N*	Event *N*	HR	95% CI	*P*-value	*N*	Event *N*	HR	95% CI	*P*-value
**Age (years)**	170	109	1.00	0.99, 1.02	.7	168	108	1.00	0.99, 1.02	.6
**Race**	170									
** White**		71	—	—						
** Asian**		14	0.76	0.43, 1.35	.3					
** Black**		9	1.27	0.63, 2.55	.5					
** Other/unknown**		15	1.14	0.65, 2.00	.6					
**Tumor location**	170					168				
** Non-upper**		82	—	—			81	—	—	
** Upper**		27	0.98	0.63, 1.51	>.9		27	1.06	0.67, 1.68	.8
**Clavien-Dindo Score**	169					168				
** Major**		7	—	—			7	—	—	
** Minor**		101	1.39	0.64, 2.99	.4		101	1.26	0.55, 2.86	.6
**Lauren type**	169					168				
** Diffuse**		41	—	—			40	—	—	
** Intestinal**		41	0.54	0.35, 0.84	**.006**		41	0.87	0.53, 1.42	.6
** Mixed**		27	0.92	0.57, 1.50	.8		27	1.26	0.74, 2.14	.4
**ypT**	170					168				
** 4**		44	—	—			43	—	—	
** 0**		1	*	*	*		1	*	*	*
** 1**		6	0.28	0.12, 0.65	**.003**		6	0.43	0.17, 1.09	.076
** 2**		13	0.37	0.20, 0.69	**.002**		13	0.53	0.26, 1.08	.081
** 3**		45	0.42	0.28, 0.64	**<.001**		45	0.60	0.36, 0.99	**.045**
**ypN**	170					168				
** N1**		31	—	—			30	—	—	
** N2**		38	1.44	0.90, 2.32	.13		38	1.38	0.85, 2.27	.2
** N3**		40	3.21	1.99, 5.17	**<.001**		40	2.34	1.37, 4.01	**.002**
**Becker TRG**	170									
** 1a/1b**		4	—	—						
** 2/3**		105	1.25	0.46, 3.41	.7					
**NAC regimen**	170									
** T + P + F**		16	—	—						
** E + P +F**		66	0.95	0.54, 1.66	.8					
** P + F**		27	1.0	0.53, 1.85	>.9					
**Post-Op LOS (days)** ^a^	170	109	1.02	0.98, 1.05	.3					
**AC receipt**	170					168				
** Non-AC**		29	—	—			29	—	—	
** AC**		80	0.63	0.41, 0.96	**.032**		79	0.75	0.47, 1.17	.2

Significant *P*-values are bolded.

*HR and CI not reported for pT0 due to a sample size of 1.

Abbreviations: E, epirubicin; F, 5FU/capecitabine; HR, hazard ratio; LN, lymph node; LOS, length of stay; LVI, lymphovascular invasion; P, platinum-based; PNI, perineural invasion; T, taxane-based; TRG, tumor regression grade.

a Postoperative LOS was evaluated using a 60-day landmark analysis.

In the ypN- cohort, there were no significant differences in EFS (median 12 vs. 10 years, *P* = .7) (Figure 2A) or OS (median 12 vs. 10 years, *P* = .5) (Figure 2B) between patients who received AC versus those who did not. Cox regression analyses of EFS and OS in the ypN- cohort are summarized in Tables S6 and S7.

**Figure 2.**
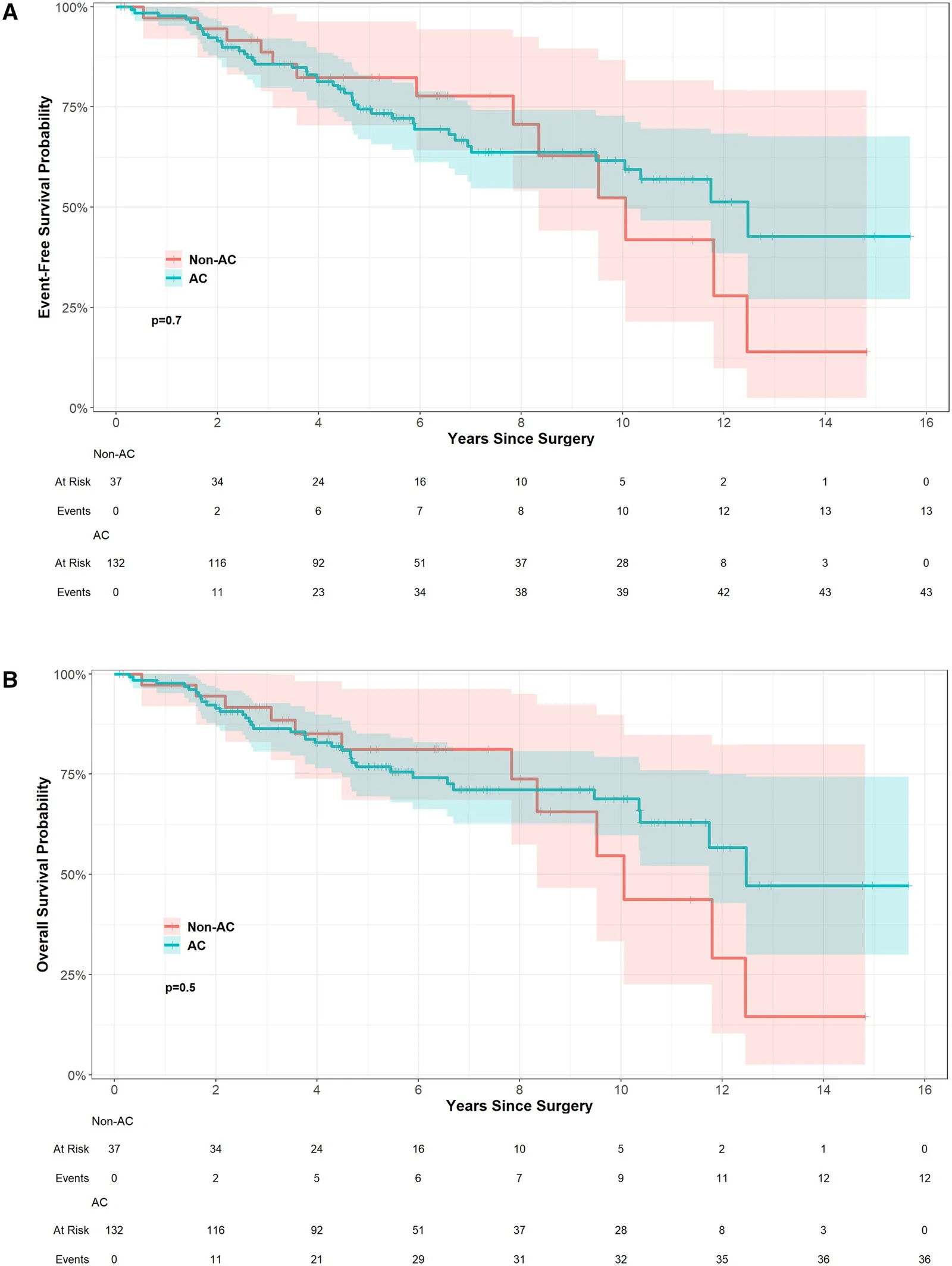
Kaplan-Meier analyses of (A) event-free and (B) overall survival among ypN− patients.

### Subgroup analysis by LNR

To further explore the potential association between AC and improved outcomes in the ypN + cohort, we performed subgroup analyses based on the lymph node ratio (LNR), the number of positive nodes relative to the number of total resected nodes.^10^ An optimally derived cutoff for LNR of 6.85% was used to divide the overall cohort into low LNR (*n* = 226; 67%) and high LNR (*n* = 112; 33%) subgroups. LNR was not available for 1 case as no lymph nodes were dissected. In both subgroups, the ypN stage distribution was similar between the AC and non-AC groups (*P* > .05). Within the high LNR subgroup, patients who received AC (*n* = 87; 78%) had significantly longer EFS (*P* = .014) and OS (*P* = .033) versus those who did not (*n* = 25; 22%) (Figure S4, see online supplementary material for a color version of this figure). Within the low LNR subgroup, there were no significant differences in EFS (*P* = .7) or OS (*P* = .6) between patients who received AC (*n* = 177; 78%) versus those who did not (*n* = 49; 22%) (Figure S5, see online supplementary material for a color version of this figure).

### Subgroup analysis by TRG

To explore the potential benefit of AC based on pathologic response to NAC, we performed subgroup analyses based on Becker TRG (1a-1b vs. 2-3). The receipt of AC was associated with improvement in EFS (*P* = .014) and OS (*P* = .024) in patients with Becker TRG 2-3 tumors, but not in patients with Becker TRG 1a-1b tumors (*P* = .5 for both EFS and OS), though the latter analysis was limited by small sample size (*n* = 45) (Figures S6 and S7, see online supplementary material for a color version of these figures).

## Discussion

In this single-center retrospective analysis of 339 patients, we found that the receipt of AC was not associated with longer survival in patients with ypN − disease. While the receipt of AC was associated with significantly longer EFS in patients with ypN + disease on univariable analysis, it was not independently associated with longer EFS on multivariable analyses. This may be due to potential confounding factors that are unaccounted for in this analysis. For example, it is possible that patients with ypN + disease who received AC were inherently fitter than those who did not receive AC. This fitness discrepancy would be supported by the imbalances we found in baseline characteristics, such as AC patients being younger and having shorter postoperative LOS. AC patients also had fewer dissected nodes which, while not routinely taken into account when making the decision of whether or not to administer AC, is another baseline difference worth noting.

Based on our analysis, we are unable to definitively determine whether AC confers a survival benefit for patients with ypN + disease. However, patients with high LNR had significantly longer EFS and OS with receipt of AC. This supports further exploration into using LNR in patients with ypN + tumors to gain important predictive information.

Several studies have previously investigated the benefit of AC following NAC but yielded discordant conclusions. Mirza et al.^11^ found that in a United Kingdom cohort of 66 patients with gastric or GEJ cancer who received NAC, 47% of patients also received AC and had longer survival than those who did not; however, this study is limited by small sample size and confounded by the high proportion (71%) of patients with ypN + disease. An analysis of 5824 patients with resectable GC from the Netherlands Cancer Registry demonstrated longer OS in patients who underwent NAC and received AC versus those who did not.^12^ In contrast, an analysis of 3449 patients from the U.S. National Cancer Database found no significant difference in OS between those who received AC and those who did not.^9^ In all 3 studies, chemotherapy-specific data, including total neoadjuvant intent, regimen, number of cycles planned, and number of cycles completed, were unavailable. Finally, a Chinese multicenter retrospective analysis of 353 patients found that after PSM, the receipt of AC was associated with longer OS in the overall cohort and in patients with LNR >9%.^10^ This study also included a Western validation cohort of 108 patients but after PSM, AC was not associated with longer OS in this cohort.

Previous studies have studied prognostic indicators of survival in patients with resected GC. Smyth et al.^5^ found that among patients enrolled to the MAGIC study, ypN + and Mandard TRG^16^ were significantly associated with OS on univariate analysis, and ypN + but not TRG remained independently associated with OS on multivariate analysis. Together with our present study (Table S5), these data suggest that the presence of lymph node metastases at surgery is a major predictor of micrometastatic disease and likelihood of recurrence.

Others have studied the benefit of AC based on pathologic response to NAC. For example, SPACE-FLOT^17^ was a real-world cohort study of 1887 patients with locally advanced GC who received neoadjuvant FLOT. Approximately half of the patients had cN + disease. Patients were trichotomized by TRG into minimal pathologic response (MR), partial pathologic response (PR), and complete pathologic response (CR). PR was associated with a significant survival benefit in favor of adjuvant FLOT, while CR and MR were not. In contrast with these findings, our present data suggest that incomplete/poor response to NAC (TRG 2/3) is associated with benefit from AC (Figures S6 and S7, see online supplementary material for a color version of these figures). In this study, poor pathologic response to NAC was the primary reason cited for ∼15% of patients who did not ultimately initiate AC, and our results do not support this practice.

The observation that AC may benefit patients whose tumors are ypN + and/or have poor pathologic response is somewhat counterintuitive. An a priori supposition is that a lack of response to NAC implies chemoresistance in micrometastatic disease so that additional adjuvant treatment with the same chemotherapy regimen will be futile. However, our observations do not support this hypothesis and suggest that poor pathologic response does not necessarily preclude potential benefit from AC. A plausible explanation is that ypN + may represent a subgroup with greater disease burden, and therefore, a higher probability of micrometastases incompletely eliminated by neoadjuvant therapy. Given the retrospective and exploratory nature of these analyses, these findings should be considered hypothesis-generating.

The present analysis is unique in its rigorous application of clinically relevant inclusion/exclusion criteria (Figure S1, see online supplementary material for a color version of this figure). For example, patients with known dMMR/MSI-H tumors were excluded on the basis of chemoresistance demonstrated in preclinical^18^ and clinical studies^19^ and because the NCCN guidelines^7^ now advise considering perioperative immune checkpoint inhibition^20^^,^^21^ for those patients. Cancers originating at the GEJ were excluded because published data from our center show worse outcomes when compared to GC, suggesting potential differences in tumor biology.^22^

This study has several limitations. Its single-center and retrospective nature preclude definitive guidance on who should and should not receive AC. Given the study design, there is clear selection bias with regard to patients who received and did not receive AC, with differences in inherent fitness, rates of postoperative complications, and pathologic response to NAC, among other confounding factors that we were unable to control for. Given the small sample size of our study, the lack of significant association between receipt of AC and survival in the multivariable analysis of the ypN + cohort may be due to insufficient statistical power. In addition, due to the retrospective nature of the study, we are unable to rule out bias with respect to the timing of follow-up scans, which could impact the determination of EFS. Finally, the addition of durvalumab to FLOT chemotherapy recently became standard of care in the perioperative setting for resectable GC.^23^ Because the patients in this study were treated before this, our findings cannot establish whether the relative contribution of postoperative chemotherapy is altered in the setting of concurrent immune checkpoint inhibition. Nevertheless, the question remains clinically relevant because in practice, postoperative chemotherapy is often delayed or omitted due to slow postoperative recovery or cumulative chemotherapy toxicity, while durvalumab is continued.

In summary, among patients with resected GC who received NAC, the analysis in this retrospective study did not demonstrate a survival difference between those who received AC and those who did not. However, subgroup analyses suggest survival benefit associated with AC in patients whose tumors are characterized by ypN+ (and in particular, high LNR) and/or TRG 2/3. These observations strongly merit further investigation.

## Supplementary Material

oyag384_Supplementary_Data

## Data Availability

Data will be made available by the corresponding authors upon reasonable request.
