# Supplementary material for: Re-evaluating the role of adjuvant chemotherapy after neoadjuvant chemotherapy in resected gastric cancer
Source: Oncologist. 2026 Oct 3;31(11):oyag384. doi: 10.1093/oncolo/oyag384 (PMC13668346; doi:10.1093/oncolo/oyag384)
Supplement: Supplementary file 1 [file oyag384_supplementary_data.docx]

**Table S1.** Primary reason for any cessation/non-initiation of adjuvant chemotherapy, and patients’ neoadjuvant and adjuvant chemotherapy regimens.

| **Characteristic** | **Overall**  **N = 339** | **Non-AC**  **N = 75** | **AC**  **N = 264** |
| --- | --- | --- | --- |
| **Reason for AC Non-Initiation** |  |  |  |
| Experiencing Toxicity | NA | 7 (9.3%) | NA |
| Postoperative Complication | NA | 15 (20.0%) | NA |
| Minimal Pathologic Response | NA | 11 (14.7%) | NA |
| No Additional Benefit per Physician | NA | 6 (8.0%) | NA |
| Pathologic CR or near-CR | NA | 5 (6.7%) | NA |
| Patient Declined | NA | 11 (14.7%) | NA |
| Poor Performance Status | NA | 12 (16.0%) | NA |
| Unknown | NA | 8 (10.7%) | NA |
| **Reason for AC Cessation / Non-Completion** |  |  |  |
| Completed | NA | NA | 208 (78.8%) |
| Disease Recurrence | NA | NA | 2 (0.8%) |
| Experiencing Toxicity | NA | NA | 36 (13.6%) |
| Postoperative Complication | NA | NA | 5 (1.9%) |
| Patient Declined | NA | NA | 2 (0.8%) |
| Unknown | NA | NA | 11 (4.2%) |
| **Neoadjuvant Regimen** |  |  |  |
| Fluoropyrimidine + Platinum + Taxane | 66 (19%) | 16 (21%) | 50 (19%) |
| Anthracycline + Platinum + Fluoropyrimidine | 189 (56%) | 26 (35%) | 163 (62%) |
| Fluoropyrimidine + Platinum | 84 (25%) | 33 (44%) | 51 (19%) |
| **Adjuvant Regimen** |  |  |  |
| Anthracycline + Platinum + Fluoropyrimidine | 115 (34%) | NA | 115 (44%) |
| Fluoropyrimidine | 30 (8.8%) | NA | 30 (11%) |
| Fluoropyrimidine + Platinum | 68 (20%) | NA | 68 (26%) |
| Fluoropyrimidine + Platinum + Taxane | 38 (11%) | NA | 38 (14%) |
| Fluoropyrimidine + Taxane | 1 (0.3%) | NA | 1 (0.4%) |
| No Adjuvant | 75 (22%) | 75 (100%) | NA |
| Other | 12 (3.5%) | NA | 12 (4.5%) |

NA = not applicable (adjuvant regimen categories do not apply to the Non-AC group); AC = adjuvant chemotherapy; CR = complete response.

**Table S2.** Univariable and multivariable analyses of event-free survival in the entire cohort

| **Characteristic** | **Univariable Analysis** | | | | | **Multivariable Analysis** | | | | |
| --- | --- | --- | --- | --- | --- | --- | --- | --- | --- | --- |
|  | **N** | **Event N** | **HR** | **95% CI** | **p-value** | **N** | **Event N** | **HR** | **95% CI** | **p-value** |
| **Age** | 339 | 165 | 1.00 | 0.99, 1.01 | >0.9 |  |  |  |  |  |
| **Race** | 339 |  |  |  |  |  |  |  |  |  |
| White |  | 115 | — | — |  |  |  |  |  |  |
| Asian |  | 17 | 0.66 | 0.39, 1.09 | 0.11 |  |  |  |  |  |
| Black |  | 17 | 1.17 | 0.70, 1.95 | 0.5 |  |  |  |  |  |
| Other/Unknown |  | 16 | 1.29 | 0.76, 2.17 | 0.3 |  |  |  |  |  |
| **Tumor Location** | 339 |  |  |  |  |  |  |  |  |  |
| Non-upper |  | 128 | — | — |  |  |  |  |  |  |
| Upper |  | 37 | 0.86 | 0.60, 1.25 | 0.4 |  |  |  |  |  |
| **Clavien-Dindo Score** | 338 |  |  |  |  |  |  |  |  |  |
| Major |  | 11 | — | — |  |  |  |  |  |  |
| Minor |  | 153 | 1.46 | 0.79, 2.69 | 0.2 |  |  |  |  |  |
| **Lauren Histologic Type** | 331 |  |  |  |  | 331 |  |  |  |  |
| Diffuse |  | 60 | — | — |  |  | 60 | — | — |  |
| Intestinal |  | 65 | 0.61 | 0.43, 0.86 | **0.005** |  | 65 | 0.91 | 0.62, 1.33 | 0.6 |
| Mixed |  | 38 | 1.02 | 0.68, 1.53 | >0.9 |  | 38 | 1.28 | 0.84, 1.96 | 0.3 |
| **ypT** | 339 |  |  |  |  | 331 |  |  |  |  |
| 0 |  | 6 | — | — |  |  | 5 | — | — |  |
| 1 |  | 10 | 0.93 | 0.34, 2.55 | 0.9 |  | 10 | 0.96 | 0.26, 3.51 | >0.9 |
| 2 |  | 20 | 2.24 | 0.90, 5.59 | 0.083 |  | 19 | 1.83 | 0.52, 6.47 | 0.3 |
| 3 |  | 72 | 3.06 | 1.33, 7.05 | **0.009** |  | 72 | 2.59 | 0.73, 9.16 | 0.14 |
| 4 |  | 57 | 6.19 | 2.67, 14.4 | **<0.001** |  | 57 | 3.98 | 1.08, 14.6 | **0.038** |
| **ypN** | 339 |  |  |  |  | 331 |  |  |  |  |
| N0 |  | 56 | — | — |  |  | 54 | — | — |  |
| N1 |  | 31 | 1.80 | 1.16, 2.80 | **0.009** |  | 31 | 1.62 | 1.02, 2.56 | **0.040** |
| N2 |  | 38 | 2.64 | 1.74, 3.99 | **<0.001** |  | 38 | 2.14 | 1.39, 3.30 | **<0.001** |
| N3 |  | 40 | 6.23 | 4.09, 9.48 | **<0.001** |  | 40 | 3.83 | 2.42, 6.07 | **<0.001** |
| **Becker TRG** | 339 |  |  |  |  | 331 |  |  |  |  |
| 1a/1b |  | 12 | — | — |  |  | 11 | — | — |  |
| 2/3 |  | 153 | 2.38 | 1.32, 4.28 | **0.004** |  | 152 | 0.90 | 0.38, 2.11 | 0.8 |
| **NAC Regimen** | 339 |  |  |  |  |  |  |  |  |  |
| Taxane-based + Platinum-based + 5FU/Capecitabine |  | 26 | — | — |  |  |  |  |  |  |
| Epirubicin + Platinum-based + 5FU/Capecitabine |  | 98 | 0.83 | 0.53, 1.30 | 0.4 |  |  |  |  |  |
| Platinum-based + 5FU/Capecitabine |  | 41 | 1.07 | 0.66, 1.76 | 0.8 |  |  |  |  |  |
| **Post-Op LOS** | 339 | 165 | 1.01 | 0.99, 1.04 | 0.3 |  |  |  |  |  |
| **AC Receipt** | 339 |  |  |  |  | 331 |  |  |  |  |
| Non-AC |  | 42 | — | — |  |  | 42 | — | — |  |
| AC |  | 123 | 0.74 | 0.52, 1.05 | 0.091 |  | 121 | 0.72 | 0.50, 1.03 | 0.074 |

Abbreviations: CI = Confidence Interval, HR = Hazard Ratio

**Table S3.** Univariable and multivariable analyses of overall survival in the entire cohort

| **Characteristic** | **Univariable Analysis** | | | | | **Multivariable Analysis** | | | | |
| --- | --- | --- | --- | --- | --- | --- | --- | --- | --- | --- |
|  | **N** | **Event N** | **HR** | **95% CI** | **p-value** | **N** | **Event N** | **HR** | **95% CI** | **p-value** |
| **Age** | 339 | 145 | 1.00 | 0.99, 1.02 | 0.6 |  |  |  |  |  |
| **Race** | 339 |  |  |  | **0.036** | 339 |  |  |  |  |
| White |  | 106 | — | — |  |  | 106 | — | — |  |
| Asian |  | 11 | 0.46 | 0.25, 0.86 | **0.014** |  | 11 | 0.41 | 0.22, 0.77 | **0.005** |
| Black |  | 15 | 1.13 | 0.65, 1.93 | 0.7 |  | 15 | 1.62 | 0.92, 2.83 | 0.092 |
| Other/Unknown |  | 13 | 1.14 | 0.64, 2.03 | 0.7 |  | 13 | 0.59 | 0.31, 1.10 | 0.10 |
| **Tumor Location** | 339 |  |  |  | >0.9 |  |  |  |  |  |
| Non-upper |  | 109 | — | — |  |  |  |  |  |  |
| Upper |  | 36 | 0.99 | 0.68, 1.44 | >0.9 |  |  |  |  |  |
| **Clavien-Dindo Score** | 338 |  |  |  | 0.5 |  |  |  |  |  |
| Major |  | 11 | — | — |  |  |  |  |  |  |
| Minor |  | 133 | 1.25 | 0.67, 2.31 | 0.5 |  |  |  |  |  |
| **Lauren Histologic Type** | 331 |  |  |  | 0.065 |  |  |  |  |  |
| Diffuse |  | 49 | — | — |  |  |  |  |  |  |
| Intestinal |  | 61 | 0.70 | 0.48, 1.02 | 0.061 |  |  |  |  |  |
| Mixed |  | 33 | 1.08 | 0.69, 1.68 | 0.7 |  |  |  |  |  |
| **ypT** | 339 |  |  |  | **<0.001** | 339 |  |  |  |  |
| 0 |  | 6 | — | — |  |  | 6 | — | — |  |
| 1 |  | 9 | 0.83 | 0.29, 2.33 | 0.7 |  | 9 | 0.87 | 0.24, 3.12 | 0.8 |
| 2 |  | 20 | 2.22 | 0.89, 5.52 | 0.088 |  | 20 | 1.99 | 0.59, 6.73 | 0.3 |
| 3 |  | 64 | 2.68 | 1.16, 6.20 | **0.021** |  | 64 | 2.26 | 0.66, 7.77 | 0.2 |
| 4 |  | 46 | 4.90 | 2.09, 11.5 | **<0.001** |  | 46 | 3.43 | 0.97, 12.2 | 0.057 |
| **ypN** | 339 |  |  |  | **<0.001** | 339 |  |  |  |  |
| N0 |  | 48 | — | — |  |  | 48 | — | — |  |
| N1 |  | 28 | 1.88 | 1.18, 3.00 | **0.008** |  | 28 | 1.60 | 0.98, 2.61 | 0.059 |
| N2 |  | 33 | 2.64 | 1.69, 4.12 | **<0.001** |  | 33 | 2.59 | 1.62, 4.13 | **<0.001** |
| N3 |  | 36 | 6.42 | 4.10, 10.0 | **<0.001** |  | 36 | 4.86 | 2.92, 8.06 | **<0.001** |
| **Becker TRG** | 339 |  |  |  | **0.009** | 339 |  |  |  |  |
| 1a/1b |  | 12 | — | — |  |  | 12 | — | — |  |
| 2/3 |  | 133 | 2.05 | 1.13, 3.70 | **0.017** |  | 133 | 0.93 | 0.39, 2.20 | 0.9 |
| **NAC Regimen** | 339 |  |  |  | 0.13 |  |  |  |  |  |
| Taxane-based + Platinum-based + 5FU/Capecitabine |  | 14 | — | — |  |  |  |  |  |  |
| Epirubicin + Platinum-based + 5FU/Capecitabine |  | 94 | 1.54 | 0.87, 2.73 | 0.14 |  |  |  |  |  |
| Platinum-based + 5FU/Capecitabine |  | 37 | 1.84 | 0.99, 3.40 | 0.054 |  |  |  |  |  |
| **Post-Op LOS** | 339 | 145 | 1.02 | 0.99, 1.04 | 0.3 |  |  |  |  |  |
| **AC Receipt** | 339 |  |  |  | 0.12 | 339 |  |  |  |  |
| Non-AC |  | 37 | — | — |  |  | 37 | — | — |  |
| AC |  | 108 | 0.74 | 0.51, 1.07 | 0.11 |  | 108 | 0.71 | 0.49, 1.05 | 0.083 |
| Abbreviations: CI = Confidence Interval, HR = Hazard Ratio | | | | | | | | | | |

**Table S4.** Characteristics of propensity score-matched cohort

| **Characteristic** | **AC** N = 70*^1^* | **Non-AC** N = 70*^1^* | **p-value***^2^* |
| --- | --- | --- | --- |
| **Age (10 years)** | 6.73 (5.71, 7.43) | 6.83 (6.08, 7.54) | 0.5 |
| **ypStage** |  |  | 0.7 |
| 0 | 10 (14%) | 8 (11%) |  |
| I | 11 (16%) | 14 (20%) |  |
| II | 25 (36%) | 20 (29%) |  |
| III | 24 (34%) | 28 (40%) |  |
| **Location** |  |  | 0.7 |
| Non-upper | 45 (64%) | 47 (67%) |  |
| Upper | 25 (36%) | 23 (33%) |  |
| **Clavien-Dindo Score** |  |  | 0.8 |
| Major | 8 (11%) | 9 (13%) |  |
| Minor | 62 (89%) | 61 (87%) |  |
| *^1^* Median (Q1, Q3); n (%) | | | |
| *^2^* Wilcoxon rank sum test; Pearson’s Chi-squared test | | | |

**Table S5.** Univariable and multivariable analyses of overall survival in the ypN+ cohort

| **Characteristic** | **Node Positive UVA** | | | | | **Node Positive MVA** | | | | |
| --- | --- | --- | --- | --- | --- | --- | --- | --- | --- | --- |
|  | **N** | **Event N** | **HR***^1^* | **95% CI***^1^* | **p-value** | **N** | **Event N** | **HR***^1^* | **95% CI***^1^* | **p-value** |
| **Age** | 170 | 97 | 1.00 | 0.98, 1.01 | >0.9 |  |  |  |  |  |
| **Race** | 170 |  |  |  |  |  |  |  |  |  |
| White |  | 66 | — | — |  |  |  |  |  |  |
| Asian |  | 9 | 0.53 | 0.26, 1.06 | 0.072 |  |  |  |  |  |
| Black |  | 9 | 1.35 | 0.67, 2.71 | 0.4 |  |  |  |  |  |
| Other/Unknown |  | 13 | 1.08 | 0.59, 1.96 | 0.8 |  |  |  |  |  |
| **Tumor Location** | 170 |  |  |  |  |  |  |  |  |  |
| Non-upper |  | 72 | — | — |  |  |  |  |  |  |
| Upper |  | 25 | 1.03 | 0.65, 1.62 | >0.9 |  |  |  |  |  |
| **Clavien-Dindo Score** | 169 |  |  |  |  |  |  |  |  |  |
| Major |  | 7 | — | — |  |  |  |  |  |  |
| Minor |  | 89 | 1.21 | 0.56, 2.62 | 0.6 |  |  |  |  |  |
| **Lauren Histologic Type** | 169 |  |  |  |  | 169 |  |  |  |  |
| Diffuse |  | 34 | — | — |  |  | 34 | — | — |  |
| Intestinal |  | 38 | 0.61 | 0.38, 0.97 | **0.037** |  | 38 | 0.91 | 0.54, 1.53 | 0.7 |
| Mixed |  | 25 | 1.02 | 0.61, 1.71 | >0.9 |  | 25 | 1.31 | 0.75, 2.27 | 0.3 |
| **ypT** | 170 |  |  |  |  | 169 |  |  |  |  |
| 4 |  | 36 | — | — |  |  | 36 | — | — |  |
| 0 |  | 1 | * | * | * |  | 1 | * | * | * |
| 1 |  | 5 | 0.28 | 0.11, 0.73 | **0.009** |  | 5 | 0.43 | 0.15, 1.20 | 0.11 |
| 2 |  | 14 | 0.49 | 0.27, 0.92 | **0.026** |  | 14 | 0.65 | 0.33, 1.28 | 0.2 |
| 3 |  | 41 | 0.47 | 0.30, 0.73 | **<0.001** |  | 41 | 0.66 | 0.39, 1.12 | 0.12 |
| **ypN** | 170 |  |  |  |  | 169 |  |  |  |  |
| N1 |  | 28 | — | — |  |  | 28 | — | — |  |
| N2 |  | 33 | 1.38 | 0.83, 2.29 | 0.2 |  | 33 | 1.30 | 0.77, 2.18 | 0.3 |
| N3 |  | 36 | 3.17 | 1.92, 5.24 | **<0.001** |  | 36 | 2.40 | 1.37, 4.21 | **0.002** |
| **Becker TRG** | 170 |  |  |  |  |  |  |  |  |  |
| 1a/1b |  | 4 | — | — |  |  |  |  |  |  |
| 2/3 |  | 93 | 1.09 | 0.40, 2.96 | 0.9 |  |  |  |  |  |
| **NAC Regimen** | 170 |  |  |  |  |  |  |  |  |  |
| Taxane-based + Platinum-based + 5FU/Capecitabine |  | 9 | — | — |  |  |  |  |  |  |
| Epirubicin + Platinum-based + 5FU/Capecitabine |  | 61 | 1.58 | 0.78, 3.21 | 0.2 |  |  |  |  |  |
| Platinum-based + 5FU/Capecitabine |  | 27 | 1.76 | 0.83, 3.75 | 0.14 |  |  |  |  |  |
| **AC Receipt** | 170 |  |  |  |  | 169 |  |  |  |  |
| Non-AC |  | 25 | — | — |  |  | 25 | — | — |  |
| AC |  | 72 | 0.66 | 0.42, 1.04 | 0.073 |  | 72 | 0.78 | 0.49, 1.25 | 0.3 |
| *^1^* HR = Hazard Ratio, CI = Confidence Interval, *HR and CI not reported for pT0 due to a sample size of 1 | | | | | | | | | | |

**Table S6.** Univariable and multivariable analyses of event-free survival in the ypN- cohort

| **Characteristic** | **Univariable Analysis** | | | | | **Multivariable Analysis** | | | | |
| --- | --- | --- | --- | --- | --- | --- | --- | --- | --- | --- |
|  | **N** | **Event N** | **HR** | **95% CI** | **p-value** | **N** | **Event N** | **HR** | **95% CI** | **p-value** |
| **Age** | 169 | 56 | 1.00 | 0.98, 1.03 | >0.9 | 169 | 56 | 1.00 | 0.97, 1.02 | 0.8 |
| **Race** | 169 |  |  |  |  |  |  |  |  |  |
| White |  | 44 | — | — |  |  |  |  |  |  |
| Asian |  | 3 | 0.35 | 0.11, 1.13 | 0.080 |  |  |  |  |  |
| Black |  | 8 | 1.31 | 0.62, 2.80 | 0.5 |  |  |  |  |  |
| Other/Unknown |  | 1 | 0.37 | 0.05, 2.70 | 0.3 |  |  |  |  |  |
| **Tumor Location** | 169 |  |  |  |  | 169 |  |  |  |  |
| Non-upper |  | 46 | — | — |  |  | 46 | — | — |  |
| Upper |  | 10 | 0.69 | 0.35, 1.36 | 0.3 |  | 10 | 0.64 | 0.32, 1.29 | 0.2 |
| **Clavien-Dindo Score** | 169 |  |  |  |  | 169 |  |  |  |  |
| Major |  | 4 | — | — |  |  | 4 | — | — |  |
| Minor |  | 52 | 1.44 | 0.52, 4.00 | 0.5 |  | 52 | 1.10 | 0.37, 3.30 | 0.9 |
| **Lauren Histologic Type** | 162 |  |  |  |  |  |  |  |  |  |
| Diffuse |  | 19 | — | — |  |  |  |  |  |  |
| Intestinal |  | 24 | 0.75 | 0.41, 1.37 | 0.3 |  |  |  |  |  |
| Mixed |  | 11 | 1.15 | 0.54, 2.44 | 0.7 |  |  |  |  |  |
| **ypT** | 169 |  |  |  |  | 169 |  |  |  |  |
| 0 |  | 5 | — | — |  |  | 5 | — | — |  |
| 1 |  | 4 | 0.57 | 0.15, 2.13 | 0.4 |  | 4 | 0.54 | 0.14, 2.06 | 0.4 |
| 2 |  | 7 | 1.67 | 0.53, 5.26 | 0.4 |  | 7 | 1.83 | 0.56, 5.95 | 0.3 |
| 3 |  | 27 | 2.99 | 1.15, 7.78 | **0.025** |  | 27 | 3.12 | 1.15, 8.44 | **0.025** |
| 4 |  | 13 | 3.59 | 1.27, 10.1 | **0.016** |  | 13 | 3.67 | 1.27, 10.6 | **0.016** |
| **Becker TRG** | 169 |  |  |  |  |  |  |  |  |  |
| 1a/1b |  | 8 | — | — |  |  |  |  |  |  |
| 2/3 |  | 48 | 1.82 | 0.86, 3.85 | 0.12 |  |  |  |  |  |
| **NAC Regimen** | 169 |  |  |  |  |  |  |  |  |  |
| Taxane-based + Platinum-based + 5FU/Capecitabine |  | 10 | — | — |  |  |  |  |  |  |
| Epirubicin + Platinum-based + 5FU/Capecitabine |  | 32 | 0.55 | 0.26, 1.16 | 0.12 |  |  |  |  |  |
| Platinum-based + 5FU/Capecitabine |  | 14 | 0.97 | 0.42, 2.20 | >0.9 |  |  |  |  |  |
| **Post-Op LOS** | 169 | 56 | 1.01 | 0.98, 1.05 | 0.5 |  |  |  |  |  |
| **AC Receipt** | 169 |  |  |  |  | 169 |  |  |  |  |
| Non-AC |  | 13 | — | — |  |  | 13 | — | — |  |
| AC |  | 43 | 0.90 | 0.48, 1.68 | 0.7 |  | 43 | 0.70 | 0.35, 1.41 | 0.3 |
| Abbreviations: CI = Confidence Interval, HR = Hazard Ratio | | | | | | | | | | |

**Table S7.** Univariable and multivariable analyses of overall survival in the ypN- cohort

| **Characteristic** | **Univariable Analysis** | | | | | **Multivariable Analysis** | | | | |
| --- | --- | --- | --- | --- | --- | --- | --- | --- | --- | --- |
|  | **N** | **Event N** | **HR** | **95% CI** | **p-value** | **N** | **Event N** | **HR** | **95% CI** | **p-value** |
| **Age** | 169 | 48 | 1.03 | 1.00, 1.06 | 0.075 | 169 | 48 | 1.02 | 0.99, 1.05 | 0.2 |
| **Race** | 169 |  |  |  |  |  |  |  |  |  |
| White |  | 40 | — | — |  |  |  |  |  |  |
| Asian |  | 2 | 0.25 | 0.06, 1.05 | 0.058 |  |  |  |  |  |
| Black |  | 6 | 1.09 | 0.46, 2.58 | 0.8 |  |  |  |  |  |
| Other/Unknown |  | 0 | 0.00 | 0.00, Inf | >0.9 |  |  |  |  |  |
| **Tumor Location** | 169 |  |  |  |  | 169 |  |  |  |  |
| Non-upper |  | 37 | — | — |  |  | 37 | — | — |  |
| Upper |  | 11 | 0.95 | 0.48, 1.86 | 0.9 |  | 11 | 0.88 | 0.44, 1.75 | 0.7 |
| **Clavien-Dindo Score** | 169 |  |  |  |  | 169 |  |  |  |  |
| Major |  | 4 | — | — |  |  | 4 | — | — |  |
| Minor |  | 44 | 1.13 | 0.40, 3.17 | 0.8 |  | 44 | 0.93 | 0.31, 2.79 | 0.9 |
| **Lauren Histologic Type** | 162 |  |  |  |  |  |  |  |  |  |
| Diffuse |  | 15 | — | — |  |  |  |  |  |  |
| Intestinal |  | 23 | 0.91 | 0.47, 1.75 | 0.8 |  |  |  |  |  |
| Mixed |  | 8 | 1.05 | 0.44, 2.51 | >0.9 |  |  |  |  |  |
| **ypT** | 169 |  |  |  |  | 169 |  |  |  |  |
| 0 |  | 5 | — | — |  |  | 5 | — | — |  |
| 1 |  | 4 | 0.57 | 0.15, 2.11 | 0.4 |  | 4 | 0.59 | 0.15, 2.22 | 0.4 |
| 2 |  | 6 | 1.39 | 0.42, 4.58 | 0.6 |  | 6 | 1.44 | 0.43, 4.90 | 0.6 |
| 3 |  | 23 | 2.51 | 0.95, 6.61 | 0.063 |  | 23 | 2.54 | 0.92, 6.99 | 0.072 |
| 4 |  | 10 | 2.65 | 0.90, 7.79 | 0.077 |  | 10 | 2.99 | 0.99, 9.03 | 0.052 |
| **Becker TRG** | 169 |  |  |  |  |  |  |  |  |  |
| 1a/1b |  | 8 | — | — |  |  |  |  |  |  |
| 2/3 |  | 40 | 1.50 | 0.70, 3.22 | 0.3 |  |  |  |  |  |
| **NAC Regimen** | 169 |  |  |  |  |  |  |  |  |  |
| Taxane-based + Platinum-based + 5FU/Capecitabine |  | 5 | — | — |  |  |  |  |  |  |
| Epirubicin + Platinum-based + 5FU/Capecitabine |  | 33 | 1.28 | 0.48, 3.36 | 0.6 |  |  |  |  |  |
| Platinum-based + 5FU/Capecitabine |  | 10 | 1.51 | 0.51, 4.43 | 0.5 |  |  |  |  |  |
| **AC Receipt** | 169 |  |  |  |  | 169 |  |  |  |  |
| Non-AC |  | 12 | — | — |  |  | 12 | — | — |  |
| AC |  | 36 | 0.80 | 0.42, 1.55 | 0.5 |  | 36 | 0.77 | 0.37, 1.60 | 0.5 |
| Abbreviations: CI = Confidence Interval, HR = Hazard Ratio | | | | | | | | | | |

**Figure S1.** Flow chart of patient selection

**Figure S2.** Event-free survival of non-AC patients/<50% planned AC cycles vs. AC patients with >= 50% of planned adjuvant therapy cycles

**Figure S3.** Overall survival stratified by AC receipt in the propensity score-matched cohort

**Figure S4.** Overall and event-free survival stratified by AC receipt in the high LNR cohort

**Figure S5.** Overall and event-free survival stratified by AC receipt in the low LNR cohort

**Figure S6.** Overall and event-free survival stratified by AC receipt in the TRG 1a/1b cohort

**Figure S7.** Overall and event-free survival stratified by AC receipt in the TRG 2/3 cohort
